# A Literature Review of Taste Change and Zinc Deficiency After Bariatric Surgery: Could There Be a Causal Link?

**DOI:** 10.1007/s11695-022-06197-4

**Published:** 2022-11-19

**Authors:** Boshra Mozaffar, Iskandar Idris

**Affiliations:** 1 MRC-Versus Arthritis Centre for Musculoskeletal Ageing Research, National Institute for Health Research Nottingham Biomedical Research Centre, Clinical, Metabolic and Molecular Physiology, University of Nottingham, Royal Derby Hospital, Uttoxeter Road, Derby, DE22 3DT UK; 2 Applied Medical Sciences, Clinical Nutrition Department, Jazan University, Jazan, Kingdom of Saudi Arabia

**Keywords:** Taste change, Taste disorder, Bariatric surgery, Zinc, Zinc sulphate or Zn, Deficiency, Supplementation, Micro-nutrient deficiencies

## Abstract

In this review, we collated evidence relating to taste change and zinc deficiency in relation to bariatric surgery (BS) and effects of zinc replacement on taste perception and speculate on the possible role of zinc deficiency to induce taste change after BS. A literature search was conducted (33 studies, *N* = 3264). We showed that taste change and zinc deficiency are frequent complications after BS, which both typically occurred at 6 months post-surgery. Our analysis did not support a causal link between the two, but similar onset of incidences indirectly indicates a link. Supplementation with 45–50 mg of zinc sulphate, higher than current recommendation, was effective in improving taste. Further studies are required to establish the causal link between the two in the context of BS.

## Introduction

Bariatric surgery (BS) has emerged as the most cost-effective treatment to help patients with obesity to lose and maintain weight [1]. Over recent decades, the incidence of BS has increased globally, with an estimated 468,609 surgeries performed in 2013 [2]. In the UK, about two-thirds of all hospital admissions in 2016 and 2017 were due to obesity. A total of 6,760 consultant sessions for BS were completed between 2016 and 2017 [3].

Taste change is a common side effect reported by patients after BS [4]. Despite this, the evidence for the incidence of taste change following BS and the mechanism for this is limited. The few studies that have examined it have provided information regarding the prevalence of taste change following BS. For example, one study has found that taste change affected 73% of patients who underwent Roux-en-Y gastric bypass (RYGBP) [5], while another study reported 82% of laparoscopic Roux-en-Y gastric bypass (LRYGB) patients and 46% of laparoscopic adjustable gastric banding (LAGB) patients experience taste change [6].

In the USA, around 200,000 patients visit doctors each year complaining of either a taste or smell change [7], while about 240,000 patients were diagnosed with taste disorders in Japan [8] in 2003. Although causes of taste change is multifactorial—e.g., radiation therapy for cancers of the head and neck; surgery to the nose, ear, and/or throat; use antibiotic of antihistamines exposure to some chemicals [7]—the exact cause of taste change after BS remains unclear. Since zinc is an important element for developing taste buds in healthy people [9] and BS can lead to reduce dietary intake as well as zinc deficiency [10], we speculate a link between zinc deficiency as a cause of taste change following BS. Due to the high risk of zinc deficiency following BS, the British Obesity and Metabolic Surgery Society (BOMSS, 2020) recommends a minimum of 2 mg of copper and a ratio of 8–15 mg zinc. The tolerable upper intake of zinc level is the maximum daily intake unlikely to cause harmful effects on health 40 mg daily for all males and females ages 19 years and above [11]. Patients who undergo biliopancreatic diversion (BPD)/duodenal switch (DS) meanwhile need higher zinc supplementation than that for SG or RYGB; the optimal level for zinc supplementation is not known but recommends starting with at least 30 mg oral zinc daily [12].

While the mechanism of taste changes following BS remains poorly understood, evidence shows that zinc deficiency causes changes in the levels of gustin concentration and salivary flow [13]. Gustin is the major zinc-containing protein in the human parotid; low levels of gustin have been linked with growth and development disturbances of the taste buds [13], and taste change therefore may be due to low levels of total parotid saliva zinc. In an animal study, authors found that zinc deficiency induces the degeneration of soft palate taste buds on microscopy observations [14]. Although many studies have reported taste changes following BS, none has discussed the effect of zinc deficiency in taste change following BS.

The aim of this review is to collate evidence on zinc deficiency and taste change following BS. Thereafter, we plan to investigate if there is a link between the two in the context of BS. This will be undertaken by discussing evidence on the association between taste change and BS, association between zinc deficiency and BS, and evidence relating to zinc supplementation on taste perception in general and specifically in relation to BS.

## Methods

### Identifying Relevant Studies (Literature Search)

A literature search was conducted using the four electronic bibliographical databases of EMBASE, PubMed, AMED, and MEDLINE. Article bibliographies were also searched and yielded additional relevant studies. The following keywords were used: taste change, taste disorder, disguise, BS, sleeve gastrectomy, gastric bypass, banding and duodenal switch, biliopancreatic diversion, zinc, zinc sulphate or Zn, deficiency, supplementation, micronutrient deficiencies, vitamin and mineral supplementation, and nutritional deficiencies. There were no restrictions on publication date to facilitate the collection and identification of all available and relevant articles published before 30 February 2021.

### Selecting Relevant Studies

**Fig. 1:**
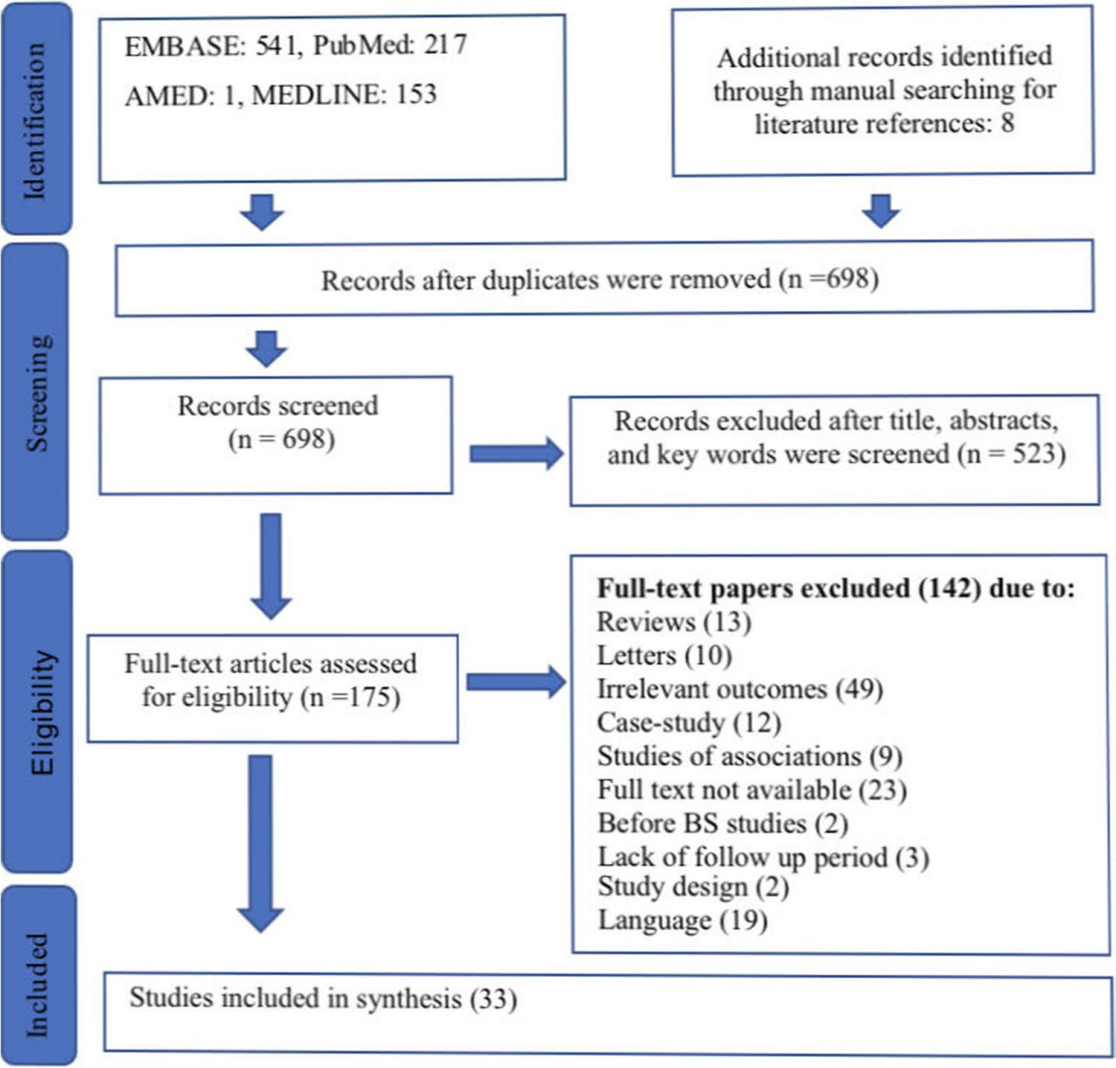
Flow diagram of study inclusions

After duplicates and irrelevant articles were removed, the literature search produced 690 articles. Of these, 523 articles were eliminated due to their irrelevance to the research question after the title and abstract were screened. The other 167 articles were assessed for eligibility, and the full texts were reviewed to determine which sources fulfilled the inclusion criteria**.** Thirty-three papers were found to fulfil the inclusion criteria (Fig. 1).

### The inclusion criteria were as follows:

1. Randomized controlled trials (RCTs) and cohort studies
2. Studies that focused on zinc deficiency and/or taste change after BS (such as sleeve gastrectomy (SG), gastric bypass (GBP), banding, duodenal switch, biliopancreatic diversion, or gastroplasty)
3. Studies populations consisting of adults ≥ 18 years
4. Studies regarding taste changes and zinc outcomes
5. Articles in the English language
6. Human studies

### The exclusion criteria were as follows:

1. Study populations ≤ 17 years
2. Studies for which the full text was unavailable
3. Studies that did not include zinc or taste change outcomes
4. Studies not in the English language
5. Cross-sectional studies and case control studies
6. Animal studies

### Data Extraction

The included studies were assessed and extracted, depending on three aspects. Table 1 describes studies about taste change following BS and classified according to author(s), year of publication, study location, study design and duration, population, type of BS, time since surgery, method, and taste change outcomes. Table 2 describes studies about zinc deficiency following BS—which are classified according to author(s), year and country, study design and duration, sample size and age, type of BS intervention and period, weight reduction science surgery, zinc dose, percentage of zinc absorption, and effectiveness of zinc supplementation. Table 3 describes the studies of the effectiveness of zinc in taste disorders. This table is classified into author(s), year and country, study design and duration, sample size, age, disease or case, methods, zinc dose/day, and treatment period.

**Table 1:** Taste change following BS

Author(s), year, and country	Study design and duration	Population	Type of BS	Time since surgery	Methods	Taste change outcomes	Food tasting differently
Burge, Schaumburg [15] Ohio	Prospective cohort; 12 weeks	*n* = 14 subjects (6 M, 8 F) Age = 38.4 ± 6 years	RYGB	6 and 12 weeks	Recognition thresholds Zinc level	Recognition thresholds for sucrose decreased significantly (*p* < .05) by 6 weeks after surgery and remained so at 12 weeks Correlation coefficients(*r* = .054) indicate no relation between taste acuity and serum zinc level	Meat, sweet taste
Zerrweck, Zurita [16] Mexico	Prospective cohort; 6 MO	*n* = 154 patients (104 LGBP/50 LSG) all females Age: 30–55 years	RYGB, SG	10 ± 6.7 MO	Taste change Survey	Increased intensity of taste, 35.5% RYGB reported taste change 34% after LSG reported taste change, *p* value = 1	Fatty food and sweet taste
Wang, [17] USA	Cohort; 1 year	*n* = 13 patients (5 M and 8 F) Age: 29–60 years	RYGB	1-month and 1-year post-surgery	fMRI and recognition thresholds	After surgery, brain activation responding to sweet tastes was significantly decreased in the reward system *p* < 0.001	Sweet taste
El Labban, Safadi [18] Lebanon	Retrospective cohort study; 6 months	*n* = 21 subjects (9 M and 12 F) Age: 19–50 years	RYGB or SG	6 MO	Sweetness acceptability test and recognition thresholds	There was no significant change in sweet sensitivity *p* > 0.05, but significant change in sourness thresholds among subjects undergoing RYGB *p* = 0.0045	Sour taste
Graham, Murty [5] Leicester, UK	Prospective cohort; 2000–2011	n = 103 patients (14 M and 89 F) Age: 25–63 years	RYGB	< 12 MO	Taste change Survey	Decreased intensity of taste; 75% reported taste changes	Meat (33%) and sweet taste
Tichansky, [6] California	Cohort	*n* = 127 patients; 82 LRYGB/28 LAGB	RYGB, LAGB	> 1MO	Taste change Survey	Decreased intensity of taste (*p* < .05) in 82% LRYGB patients and 46% LAGB patients (*p* < .001)	Sweet food and meat
Nance, Eagon [19] USA	Cohort	*n* = 31 subjects RYGB (*n* = 23)/SG (*n* = 8) Age: 26–53	RYGB, SG	NR	Detection thresholds, suprathreshold	There was no significant effect of BS on taste change *p* > 0.05	No change
Gero, Dib [20] USA^1^	Prospective longitudinal observational study; 2014–2015	*n* = 100 consecutive LSG patients(77 F and 23 M) Age: 40.8 ± 12 years	LSG	6 MO	Taste change Survey	Taste preferences for bitter, salty, umami, fatty, sour, spicy, and sweet decreased significantly from baseline to post-surgery month 6 (*p* < 0.002)	Sweet food

Summary of 8 studies

^1^*n*, number; *SD*, standard deviation; *M*, male; *F*, female; *MO*, month; *NR*, not reported; *LGBP*, laparoscopic gastric bypass; *LSG*, laparoscopic sleeve gastrectomy; *LRYGB*, laparoscopic Roux-en-Y gastric bypass; *LAGB*, laparoscopic adjustable gastric banding; *RYGB*, Roux-en-Y gastric bypass; *SG*, sleeve gastrectomy

**Table 2:** Zinc deficiency following bariatric surgery

Author(s), year, and country	Study design and duration	Sample size and age	Type of BS	Intervention and period	Weight reduction since surgery	Effectiveness of zinc supplementation	Zinc dose	% zinc absorption
Ruz, Carrasco [21] USA	RCT; August 2004 and December 2006	*n* = 67 18–55 years; F: 67	RYGBP	Group A: received the standard vitamin and mineral supplement Group B: received the improved vitamin and mineral supplement Before, 6, 12, and 18 months	27.9% 6 mo 35.2% 18 mo	Zinc deficiency significantly developed in both groups. By MO 6 90.9 ± 6 19.0 µg/dl; *p* < 0.001	Standard: 7.5 mg/d Improved: 15 mg/d	Decreased significantly from 32.3 to 13.6% at 6 mo after RYGBP and to 21% at 18 mo after surgery
Papamargaritis [22] UK	Prospective cohort; 36 months	*n* = 437 patients; 82% F; age: 45.9 ± 11.2 years	RYGB/SG/AGB 57%/20%/23%, respectively	Stander multivitamin supplementation; 3, 6, 12, 18, 24, and 36 months	NR	Zinc deficiency ranged from 7 to 15% 3 MO: 18 (9.1%) 6 MO: 24 (13.8%) 12 MO: 18 (14.8%) 18 MO: 5 (12.5%) 36 MO: 1 (2.3%)	Zinc 15 mg/d	NR
Balsa [23] Spain	Cohort; 5 years	*n* = 141 patients, 52 (RYGB) and 89 (BPD)	RYGB BPD	Stander multivitamin supplementation; followed up at 3, 6, 12, 24, 36, 48, and 60 months	NR	Zinc level significantly decreased in the BPD group (*p* < 0.0001) In RYGB zinc deficiency significantly lower at 48 and 60 months (15.4% and 21.2%, respectively)	8 mg/d	NR
Salle [24] France	Cohort; June 2005 and 2009	*n* = 324 patients 48 M and 276 F; RYGB (*n* = 266) or SG (*n* = 33) or DS (*n* = 25); age: 29–65 years	RYGB SG DS	Multivitamin supplementation; follow-up 6,12 and 24	% of weight loss 6 MO: 25.2 ± 6.6 12 MO: 32.5 ± 8.8 24 MO: 33.4 ± 11.1	% of zinc deficiency patients. 6 MO: 35.6%; 12 MO: 42.5%; 24 MO: 34.8%; zinc deficiency was present in 42.5% of patients at M12 and more frequent after DS (91.7%) at M12	15 mg/d	NR
Billeter, Probst [25] USA	Prospective cohort; 2 years	20 patients 50% male Age: 18–70 years	RYGB	Multivitamin supplementation; follow-up, 3, 6, 12, and 24	NR	Zinc decreased significantly over 24 months 13.9 ± 0.5 to 10.8 ± 0.5. mol/l	NR	NR
[26] USA	Cohort December 2002 to June 2003	*n* = 100 patients	LRYGB	Multivitamin supplementation Follow-up, 3, 6, 12	NR	Zinc deficiency 3 MO 23%; 6 MO 23%; 12 MO 36%	NR	NR
Rojas, Carrasco [27] Chile	RCT 2004–2008	*n* = 63 F; mean age: 36.9 ± 9.2 years	RYGB	Group A: received the standard vitamin and mineral supplement Group B: received the “improved vitamin and mineral supplement” Group C: extra zinc supplement; baseline and 6 months after RYGBP	NR	Plasma zinc concentration increased in all groups after 6 months of surgery: 8.6 ± 23.2 *p* = 0.007	A: 7.5 mg/d B:15 mg/d C: 25 mg/d	NR
Gobato, Seixas Chaves [28] Brazil	Cohort	*n* = 36 patients Age: 18 to 65, 75% F	RYGB	Multivitamin supplementation, baseline, and 6 months after RYGBP	The mean weight loss from baseline to 6 months after surgery was 35.34 ± 4.82%	(61.11%) patients reported zinc deficiency 6 months after surgery	7 mg/d of zinc oxide	NR
Pires, Martins [29] Brazil	Cohort	*n* = 22 patients; age 19–56 years	RYGB	No supplementation baseline and 6 months after RYGBP	Weight after 6 months of surgery 80.50 ± 12.60 kg	At 6 months after the RYGBP operation, the patients had decreased zinc concentration in urine compared with the initial value, and the difference was statistically significant (*p* < 0.05)	NR	NR
Dalcanale, Oliveira [30] Brazil	Prospective cohort; 2 years	*n* = 75 Age: 18–65 years; M: *n* = 7, F: *n* = 67	GBP	Multivitamin supplementation; 2 years	EWL% = 50% or above	40.5% of patients had zinc deficiency, which was significant (*p* = 0.008)	25 mg/d	NR
Pech, Meyer [31] Germany	Prospective, single centre, observational, 2005–2009	*n* = 82 patients underwent SG Age: 22–64 years; M: 48, F: 34	SG	33 patients underwent zinc supplementation 3, 6, 12, 18, and 24 months	EWL% was 54.3% after 6 months and 65.3% after 12 months	30 patients received zinc supplementation; 13.4% of participants had zinc deficiency after surgery	15 mg/d	NR
Katsogridaki, Tzovaras [32] Greece	Prospective cohort study; 2013–2015	50 patients; 14 M (28%) and 36 F (72%), age: 38.74 ± 11.87 years	LSG	6 months after surgery Multivitamin supplementation	NR	Zinc deficiency was 7% preoperative 0.70 (0.20) (*p* < 0.001) and ranged from 7 to 15% post-operative 0.58 (0.19) (*p* < 0.001) in patients receiving multivitamins and mineral supplementation post-operative, 0.11 95%CI (− 0.16–0.071)	7 mg/d	NR

^2^*n*, number; *SD*, standard deviation; *M*, male; *F*, female; *MO*, month; *NR*, not reported; *LGBP*, laparoscopic gastric bypass; *LSG*, laparoscopic sleeve gastrectomy; *LRYGB*, laparoscopic Roux-en-Y gastric bypass; *LAGB*, laparoscopic adjustable gastric banding; *RYGB*, Roux-en-Y gastric bypass; *SG*, sleeve gastrectomy

Summary of 12 studies

**Table 3:** Zinc and taste disorder

Author(s), year, and country	Study design and duration	Sample size	Age	Disease or case	Methods	Zinc dose/d	Treatment period	Outcomes
Khan [33] Pakistan	RCT Oct 2017 to Mar 2018	17 patients 32.4% females	46 ± 9.2, 32.4% females	Chemotherapy-related taste alteration (oral cancer)	Detection thresholds, serum zinc level	Zinc sulphate 50 mg orally 3 times/d	7–8 MO treatment 1-MO follow-up	Zinc sulphate was not found to be preventing chemoradiation induced taste alterations Sweet taste was most effected by cancer *p* = 0.04
Ripamonti [34] Italy	RCT 1995–1996	18 patients (8 F and 10 M)	Mean (SD) age 55.5 (14.39)	Head and neck cancer	Detection thresholds, serum zinc level	Zinc sulphate 45 mg orally 3 times/d	1 MO	A rapid improvement of taste was observed in 64% of patients treated with zinc, compared with 22% of the placebo group
Sakai [35]) Japan	RCT 1991–1994	73 patients 47 F	23 to 79 years	Idiopathic taste disorder	Filter paper disk method, serum zinc level	29 mg of zinc picolinate/3 times/d	3 MO	Zinc level before treatment 69 mg/dl or lower. Patients’ zinc picolinate-treated group had significantly high improvement (*x*^2^ test: *p* = 0.01) compared to placebo
Halyard, Jatoi [36] USA	RCT 2002–2005	169 patients 118 M; 51 F (Co = 84 + Int = 85)	≥ 18 years	Head and neck cancer	Taste change questionnaire + serum zinc level	Zinc sulphate 45 mg orally 3 times/d	6 MO	No significant difference in taste between zinc-treated group and placebo *p* = 0.09
Lyckholm [37] USA	RCT 2002–2005	58 cancer patients 41 females	≥ 18 years	Chemotherapy-related taste (cancer)	Taste change questionnaire + serum zinc level	50 mg of elemental zinc/2 times/d	3 MO	There was no statistically significant improvement after zinc supplementation treatment (*p* < .0001)
Mahajan, Prasad [38] USA	RCT	22 patients	51.3 ± 3.2	Uremic hypogeusia	Detection thresholds, serum zinc level	50 mg of elemental zinc as zinc acetate/d	6 MO	After 6 to 12 weeks of therapy, patients showed significant improvements in taste. The mean plasma zinc level increased from 75 ± 8 to 97 ± 10 ısg/dl (*p* < 0.001) in patients receiving zinc acetate
Sakagami [39] Japan	RCT	109 patients Placebo (*n* = 28), zinc-treated *n* = 81 (56 F and 51 M)	20–80 years	Idiopathic taste disorders	Filter paper disk method, serum zinc level	17 mg (*n* = 27), 34 mg (n_26), or 68 mg (n_28) of oral zinc, polaprezinc preparations, daily	12 weeks	Zinc level before treatment lower than 69 mg/dl The group of patients given 68 mg zinc showed a significant improvement; 25 patients cured and improved in their gustatory sensitivity, compared with the placebo group *p* = 0.018
Ikeda [40] Japan	1 month	408 patients 228 females	49–65 years	Taste disorder in elderly	Filter paper disk method, serum zinc level	Zinc agent polaprezinc 75 mg, 2 times/day, containing 17 mg of zinc	1 MO	Zinc level before treatment 69 mg/dl or lower 1 MO after treatment had significantly increased, to 91.0 mg/dl (*p*, 0.001) 35 of zinc-deficient patients were cured or improved of taste disorder
Henkin, Schecter [41] Washington	RCT	106 patients (53 M and 53 F)	19–84 years	Taste dysfunction	Detection thresholds, serum zinc level	100 mg of zinc ion in four divided oral doses	6 MO	Zinc sulphate was effective in taste treatment
Heckmann, Hujoel [42] Germany	RCT 1999 to 2001	*n* = 50 patients (7 M and 43 F)	41–82 years	Dysgeusia	Filter paper strips and serum zinc level	140 mg/d of zinc gluconate, equivalent to 20 mg/day of elemental Zinc	3 MO	Intervention group reported improvements of gustatory function after zinc treatment (*p* < 0.001) taste test—before (mean SD) (17.1, 5.8) and after (9.04, 13.04); zinc in serum before (mg/dl) = (72.78,18.38) and after (81.38,19.61), and rated dysgeusia as being less severe(*p* < 0.05)
Atkin-Thor, Goddard [43] USA	RCT	20 patients	21–70 years	Hypogeusia	Detection thresholds, serum zinc level, hair zinc	440 mg ZnSO4 Post-dialysis, 3 times per week	6 weeks	After supplementation with Zn, taste acuity markedly improved in 95% of patients and Zn concentrations in hair increased in 85% of patients. The patients’ appetites improved
Stewart-Knox, Simpson [44] Tokyo, Japan	RCT	199 healthy older F = M	70–87 years	Taste disorder in elderly	Detection thresholds, serum zinc level	15 or 30 mg Zn/d	6 MO	Serum Zn increased post-intervention, intervention. Salt taste acuity was greater in response to Zn (30 mg) supplemented group (0·84 409 (SD 0·13 349) than the placebo group (0·75 045) (SD 0·210) post-intervention, in the Grenoble
Najafizade, Hemati [45] Iran	RCT	35 patients (F 14 and M 21)	Age = 59.2 ± 16.5, 60% male	Head and neck cancers	Detection thresholds, serum zinc level	50 mg/ 3 times/d	5–9 weeks	There was a significant increase in taste perception threshold for bitter, salty, sweet and sour tastes in placebo group (*p* = 0.001). Intervention group slightly increased in threshold for perception of salty taste (*p* = 0.046)

Summary of 13 studies

^3^*n*, number; *RCT*, randomized controlled trials; *SD*, standard deviation; *M*, male; *F*, Female; *MO*, month; *Zn*, zinc; *d*, day; *mg*, milligramme; *ZnSO4*, Zinc sulphate

Only one study was found regarding the role of zinc in taste change following BS [15], during data extraction. Meanwhile, many studies were found related to the effectiveness of zinc in taste change treatment.

## Results

### Quality Assessment

The quality of the papers included was evaluated, using the Newcastle–Ottawa scale for randomized control trials and cohort studies. The quality assessment for the randomized controlled trial comprised three categories (“selection” contains four questions, “comparability” contains one question, and “exposure” contains three questions). The quality assessment for the cohort studies also included three categories (“selection” contains four questions, “comparability” contains one question, and “outcome” contains three questions). Each study can be given a maximum of one star for each numbered item within the categories. A maximum of two stars can be given for comparability. Thresholds for converting the Newcastle–Ottawa scales to AHRQ standards (good, fair, and poor) are the following: good quality, 3 or 4 stars in the selection domain AND 1 or 2 stars in the comparability domain AND 2 or 3 stars in the outcome/exposure domain; fair quality, 2 stars in the selection domain AND 1 or 2 stars in the comparability domain AND 2 or 3 stars in the outcome/exposure domain; and poor quality, 0 or 1 star in the selection domain OR 0 stars in the comparability domain OR 0 or 1 stars in the outcome/exposure domain.

#### Randomized Controlled Trial (RCT) Studies’ Quality Assessment

Twelve studies were deemed to be of good quality [33–39, 41–45]: Three studies were of fair quality [21, 27, 40] (*see Table **4*)*.*

**Table 4:** Quality assessment for 15 randomized controlled trials

Author	Selection				Comparability		Exposure			Total
	Case definition	Representativeness of case	Selection of controls	Definition of controls	Main factors	Additional factors	Ascertainment of exposure	Same method for cases and controls	Non-response rate	
Khan [33]	*	*	*	*	*	*	*	*	0	8
Najafizade, Hemati [45]	*	*	*	*	*	0	*	*	0	7
Lyckholm [37]	*	*	*	*	*	*	*	*	0	8
Rojas, Carrasco [27]	*	*	0	0	*	*	*	*	0	6
Ruz, Carrasco [21]	*	*	0	0	*	*	*	*	0	6
Sakagami [39]	*	*	*	*	*	*	*	*	0	8
Ikeda [40]	*	*	0	0	*	*	*	0	0	5
Stewart-Knox, Simpson [44]	*	0	*	*	*	0	*	*	0	6
Halyard, Jatoi [36]	*	*	*	*	*	*	*	*	*	9
Heckmann, Hujoel [42]	*	*	*	*	*	*	*	*	0	8
Sakai [35]	*	*	*	*	*	0	*	*	0	7
Ripamonti [34]	*	*	*	*	*	*	*	*	0	8
Mahajan, Prasad [38]	*	*	*	*	*	0	*	*	0	7
Atkin-Thor, Goddard [43]	*	*	*	*	*	*	*	*	0	8
Henkin, Schecter [41]	*	*	*	*	*	*	*	*	0	8

#### Cohort Studies’ Quality Assessment

Nine studies were of good quality [17, 19, 20, 22, 25, 28, 29, 31, 32]. Eight studies were of fair quality [5, 6, 16, 18, 23, 24, 26, 30]. One study was poor quality [15] (*see Table **5*).

**Table 5:** Quality assessment for 18 cohort studies

Author	Selection				Comparability		Outcome			Total
	Exposed cohort	Non-exposed cohort	Ascertainment of exposure	Outcome of interest not present at start	Main factors	Additional factors	Assessment of outcome	Follow-up long enough*	Adequacy of follow-up	
Katsogridaki, Tzovaras [32]	*	*	*	0	*	*	0	0	*	6
Nance, Eagon [19]	*	*	*	0	*	*	*	*	*	8
Gero, Dib [20]	*	*	*	0	*	0	*	*	*	7
El Labban, Safadi [18]	*	0	*	0	*	*	*	*	*	7
Zerrweck, Zurita [16]	*	0	*	0	*	*	*	*	*	7
Billeter, Probst [25]	*	0	*	*	*	*	*	*	*	8
Papamargaritis [22]	*	0	*	*	*	*	*	*	*	8
Wang, [17]	*	*	*	0	*	0	*	*	*	7
Graham, Murty [5]	*	0	*	0	*	0	*	*	*	6
Gobato, Seixas Chaves [28]	*	0	*	*	*	0	*	*	0	6
Balsa [23]	*	0	*	0	*	*	*	*	*	7
Salle [24]	*	0	*	0	*	*	*	*	*	7
Madan, Orth [26]	*	0	*	0	0	0	*	*	*	5
Burge, Schaumburg [15]	*	0	0	0	0	*	*	0	0	3
Dalcanale, Oliveira [30]	*	0	*	0	*	*	*	*	*	7
Pech, Meyer [31]	*	0	*	*	*	*	*	*	*	8
Pires, Martins [29]	*	0	*	*	*	0	*	*	0	6
Tichansky, [6]	*	0	*	0	0	0	*	0	0	3

#### Association Between Bariatric Surgery with Taste Change: Qualitative Studies

A sample of 103 patients who underwent RYGB, 75% reported taste changes in a period of less than 12 months and that the foods that most commonly tasted different were meat (33%) and sweet flavours [5]. In a comparison between BS procedures, authors found that 35.5% of RYGB and 34% of laparoscopic sleeve gastrectomy (LSG) patients reported a taste change in commonly consumed food, in a mean time of 10 ± 6.7 months after surgery [16]. While there was no significant difference between the two surgeries in term of taste change, patients presenting with food aversion experienced higher excessive weight loss percentage (%EWL) compared with those without aversion, (73.3 ± 19.7 vs 65.8 ± 19.4%; *p* = 0.046) [16]. In a further study, a comparison was made between RYGB and laparoscopic adjustable gastric banding (LAGB) in a sample of 127 patients, of whom 82 underwent RYGB and 28 underwent LAGB. Decreased intensity of taste was reported in 82% of RYGB patients and 46% of LAGB patients (*p* < 0.05), and 83% of RYGB patients and 69% of LAGB patients agreed that loss of taste led to better weight loss [6]. A recent study of 100 patients who underwent LSG found a decrease in preferences for core tastes, 6 months after surgery (*p* < 0.002); in this study, the decrease in preferences did not influence significantly the % of total body weight loss except for salty taste a higher decrease in salty preferences correlated with a higher % of total body weight loss (*p* = 0.02) [20]. In all four included studies that used questionnaires, the most important decline in taste preference was observed for sweet food [5, 6, 16, 20] (Table 1).

#### Association Between Bariatric Surgery with Changes in Sensory-Discriminative Component: Quantitative Studies

The evaluation of taste perception includes assessment of taste quality (sweet, salty, bitter, sour, umami) and taste sensitivity. The four included studies measured detection thresholds (the minimum concentration a subject must taste to identify a taste stimulus as being different from water) or recognition thresholds (the minimum concentration that a subject need to recognize the taste quality of the stimulus). Burge, Schaumburg [15] found an increase in sweet taste sensitivity (decreased thresholds for sucrose detection and recognition) at 6 weeks post-RYGB (*p* < 0.05). In contrast, Nance, Eagon [19] found no change in taste sensitivity after 1 year of LSG and RYGB *p* > 0.05. El Labban, Safadi [18] did not find a significant change in sweet sensitivity (*p* > 0.05), but did find a significant change in sourness thresholds, among subjects who had undergone RYGB *p* = 0.0045. In a pilot functional magnetic resonance imaging (fMRI) study, the authors examined taste testing and fMRI for 13 subjects pre- and post-BS and compared to controls in response to sweet and salty solutions; the author found a significant decrease in brain activation in the reward system responding to all sweet tastes compared to pre surgery; however, the same effect appears in non-surgical controls (*p* < 0.001) [17]. In contrast, significant increase in brain activation in the reward system responds to salty tastes after surgery compared to controls [17].

### Association Between Bariatric Surgery with Zinc Deficiency

Twelve studies have investigated zinc status following BS. A randomized controlled trial of zinc absorption and zinc status after RYGB found that zinc absorption decreased significantly, from 23.3 to 13.6%, during the first 6 months after surgery. Patients’ zinc levels thus diminished, despite zinc supplementation at 15 mg/day. However, there was a slight improvement of zinc absorption at the end of the study (18 months) [21]. In Papamargaritis [22]’s study, the author found that zinc deficiency doubled from 7 to 15%—before and after surgery, respectively. The percentage of zinc deficiency peaked at 6 months after surgery (*n* = 24; 13.8%). The percentage then decreased slightly throughout the follow-up period, to reach 7.1% by 36 months after surgery [22]. In contrast, Rojas, Carrasco [27] found zinc concentration levels to increase after 6 months of RYGB, despite the significant reduction in dietary intake of zinc and regardless of the supplementation group (*p* < 0.001). Ruz, Carrasco [21] and Papamargaritis [22] found in cohort studies of zinc deficiency after SG, RYGB, and duodenal switch (DS) that zinc deficiency was common in 42.5% of patients, out of a sample of 324 patients at 12 months after surgery. Zinc deficiency was more prevalence in DS patients, with a percentage of 91.7% despite zinc supplementation at 22 mg/day [24]. Another cohort study compared serum zinc levels in 52 RYGB and 89 BPD patients. Zinc levels were significantly low in both groups (*p* < 0.0001); however, zinc deficiency was more frequent in BPD patients—ranging from 44.9 to 74.9% deficiency Balsa [23]. Further studies [29] have supported the findings from Ruz, Carrasco [21] which showed that the peak effect of plasma zinc concentrations after RYGB occurred at ~ 6 months after surgery (*p* < 0.05) [21, 25, 26, 28–30]. There was however some discordance on the effects of LSG on zinc levels; i.e., Pech, Meyer [31] did not find significant zinc deficiency following LSG, while Katsogridaki, Tzovaras [32] found significant zinc deficiency after LSG (*p* < 0.001) (Table 2).

### Effectiveness of Zinc Replacement to Improve Taste Change

Thirteen studies have examined the effectiveness of zinc replacement as a treatment to taste change. All studies are randomized controlled trials, and none was performed in BS. Five studies have examined the effectiveness of 45–50 mg of zinc sulphate supplementation either 2 or 3 times per day, in cancer patients with head and neck cancer or chemotherapy, over a treatment period ranging from 1 to 8 months [33, 34, 36, 37, 45].

A study in 2019 study Khan [33] found that zinc sulphate was not effective in the treatment of taste alterations induced by chemoradiation (*p* = 0.04) [33]. In contrast, an older study conducted in 1998, authors found a significant improvement in taste—which was measured at 64% in the zinc-treated group, compared with 22% in the placebo group, after receiving 45 mg of zinc 3 times per day over a 1-month period [34]. Both authors used detection thresholds to measure taste. Halyard, Jatoi [36] introduced 45 mg of zinc sulphate 3 times per day in a sample of 169 patients with head and neck cancer for a period of 6 months. The investigator found no significant difference in taste improvement, in either the zinc-treated or the placebo group (*p* = 0.09) [36]. Similarly, Lyckholm [37] did not find a significant improvement in 58 patients after introducing 50 mg of zinc sulphate for 3 months, and both authors used a taste questionnaire to identify taste change. Najafizade, Hemati [45] found a slight increase in the threshold for the perception of salty taste (*p* = 0.046). Two studies Ikeda [40] examined the effectiveness of zinc supplementation in taste disorder treatment in elderly patients. Ikeda [40] studied a large number of participants—408 patients, who received 17 mg of zinc—and found their zinc levels to increase significantly after 1 month of the treatment, from 69 mg/dl or lower to 91.0 mg/dl (*p*, 0.001). Stewart-Knox, Simpson [44]’s study also showed significant improvements in taste after receiving 15–30 mg of zinc over 6 months.

Six studies involved participants with taste dysfunction. All these studies reported significantly high improvements in zinc levels after zinc supplementation. They found that taste dysfunction either improved or was cured—in a treatment period ranging from 3 to 6 months with a zinc dose ranging from 20 to 50 mg/day [35, 38, 39, 41–43] (Table 3).

## Discussion

Numerous studies have been carried out on taste change following BS. Investigators have used different methods to determine said taste change. In this review, we have found that the results differed depending on the methods used to assess taste change. For instance, survey studies found that a considerable percentage of patients claimed to have taste change following BS, at a mean time of 6 months post-surgery [5, 6, 16, 20], consistent with a review by Ahmed, Penney [4]. However, studies that examined taste change via experimental methods like recognition thresholds, fMRI, and the sweetness acceptability test did not find a significant change in taste following BS [18, 19]. There was however one exception, where a study found an increase in sweet taste sensitivity, 6 months after RYGB surgery [15]. It is suggested that the recognition threshold methods used in the above-mentioned studies do not reflect current changes in taste intensity, as the concentrations are more closely related to our food experiences. While the discordance between findings from self-reported compared with experimental studies may not be overtly surprising, we believe that conclusion from self-reported studies would still play an important role in investigating the effects of BS on taste change, as this may still play an important role in patients eating behaviour. A study to validate self-reported survey with experimental study would be important to resolve future discordance findings between the two.

We suggest several reasons for taste change after BS. First, it may be associated with the rate of salivary flow after BS; Marsicano, Grec [46] found a reduction in the salivary flow rate, 3 months after surgery (*p* < 0.05). Saliva is essential to dissolve food particles and stimulate taste receptor cells on the taste buds, located on the tongue papillae. Saliva dissolves some tastants, which then diffuse to the taste receptor sites. Taste sensitivity is related to the composition of saliva, in a complex process. For example, salivary bicarbonate ions can reduce the concentration of free hydrogen ions and thereby affect the sour taste. Proline‐rich proteins can affect the bitter taste. The other taste stimuli (sweet, salty, and umami) are likewise affected by different elements in saliva. Thus, reduced salivary flow affects the taste threshold following BS. Second, changing levels of gustin concentration may also induce taste changes. As noted, gustin is the major zinc-containing protein in the human parotid; changes therein may be linked with zinc deficiency. Shatzman [13] found that patients with hypogeusia had low concentrations of zinc in their saliva—administering zinc to these patients was associated with increased salivary zinc content and gustin concentrations. Third, nutritional deficiencies such as vitamin B12 deficiency which may manifest as a smooth, red tongue may lead to a loss of taste perception [47].

This review also offers insight via the evidence showing that zinc deficiency was clearly reported 6 months after BS [21, 22, 25, 26, 28–30]. One study showed paradoxical results, in which patients’ levels of zinc concentration increased after 6 months of RYGB [27]. These results should be interpreted with caution. The authors suggest that inflammation decreases zinc levels and that reduced inflammation was observed in their subjects. RYGB patients were particularly affected by zinc deficiency, because of the restrictive and malabsorptive nature of this surgery. The exclusion of the interior part of the stomach, duodenum, and proximal jejunum after RYGB causes a malabsorption of zinc—which is primarily absorbed in the duodenum—along with low dietary intake [48, 49]. Yet, in a comparison between different BS procedures, Salle (2010) found that zinc deficiency was more frequent after DS and SG than after RYGB [24]. Another cohort found that zinc deficiency was higher in BPD patients, compared to RYGB [23]; however, Ruiz-Tovar [50] found that zinc level was in normal range one year after SG; zinc = 86.9 µg/dl.

Despite differences in the prevalence of zinc deficiency following different BS procedures, zinc deficiency frequently occurs in all types of BS procedures. However, studies by Balsa [23] and Salle [24] reported that the number of patients undergoing each procedure was not equal, which may influenced the reported percentage of individuals affected by zinc deficiency [23, 24]. Furthermore, most studies(29) focused on one type of BS procedure, RYGB. They likewise studied just a small number of patients [21, 25, 26, 28–30].

Studies have reported zinc deficiency, even when multivitamins and mineral supplementation were prescribed to patients following surgery. There are several possible explanations for this. First, zinc absorption decreases significantly after surgery. Ruz, Carrasco [21] found a significantly decrease of zinc absorption from 32.3 to 13.6% at 6 months, after RYGBP, and to 21% at 18 months after surgery (see Table 2). To the best of our knowledge, no previous studies have specifically evaluated zinc absorption after BS, except from that of Ruz, Carrasco [21]. Second, the level of zinc dosage is too low to have a significant impact on absorption. It is suggested that the standard multivitamins and mineral supplementation prescribed by surgeons in current clinical practice—at between 8 and 15 mg of zinc—are not effective for avoiding zinc deficiency following surgery. Additional studies should be conducted to evaluate zinc absorption after BS.

Many high-quality papers have found a significant association between taste change and zinc deficiency. While causal link between taste and zinc deficiency after BS cannot be concluded from this review, the onset of both at approximately 6 months may suggest an indirect link between the two. Further interventional studies are required to confirm causal link. Importantly, current evidence has shown improvements in taste function, for patients with taste dysfunction, after receiving 30–45 mg of zinc supplementation daily over 6 months [35, 38–43, 45]. This is twice the dosage usually prescribed to patients following BS. This suggests that zinc is an effective treatment for taste change, in many cases of taste disorder induced by different diseases. Yet studies into taste change in cancer patients found no significant improvement after similar doses of zinc supplementation. This may be explained by several interpretations. First, a small sample size makes it difficult to detect a significant difference between the placebo and intervention groups. Second, a lack of follow-up assessment may influence the outcome. Third, the absence of a formal taste test may impact the results. Fourth, mucositis and oral infections may also influence taste changes—as may other medications taken by cancer patients—and may also be important factors in delayed zinc intervention. It is also suggested that routine zinc supplementation after BS is not effective to avoid taste change and that patients require a double dosage, based on above-mentioned studies. We therefore believe that results from this review are in agreement with another review [9], stating that zinc supplementation may potentially be used to treat taste disorders.

A high-quality RCT of 40 subjects with obesity found that 30 mg/day of zinc supplementation for 15 weeks with a restricted calorie diet of almost 300 kcal lower than the estimated energy requirements had a positive impact in weight reduction and appetite; supplemented group with zinc had lower appetite and more weight reduction comparing with placebo group which follow only restricted calorie diet [51].

This review also found that patients who experiences taste change had higher % EWL comparing with those with no taste change. In agreement with these findings, Makaronidis, Neilson [52] found a significant correlation between taste change and percent of weight loss following RYGB: 27.8%, *n* = 3, comparing to 23.1%, *n* = 35, with no taste change, confounding factors including procedure and patient’s selection and questionnaire design may have influence these findings [52]. Further studies on the relationship between taste change and % EWL with comparison between different procedures are needed. A recent study suggests that subjects with high % EWL had a significantly decrease preference to sweet-tasting food following surgery [53]. Taste alteration also has been linked to ageing. Around the age of 60, the ability to taste gradually decreased [54]. In this review, elderly people with taste disorder showed improvements of taste sensitivity after receiving zinc supplementation [40, 44]. However, the age-related taste change after bariatric surgery has not previously been described in the current studies. This may constitute the object of future studies.

### Strengths and Limitations

This review objectively examines the effects of zinc deficiency on taste change after BS. Several limitations need to be highlighted which may influence the interpretation derived from studies obtained from this review. One of the limitations of this study is the heterogeneity of the studies’ design, including most of the cohort and RCT studies. Another limitation is the lack of studies directly examining the effect of zinc on taste change, following BS. Lastly there are some caveats to interpreting circulating zinc level. All the studies measure plasma/serum level of zinc. While this may provide marker of patients circulating zinc level, it may not accurately reflect patients zinc status, especially in non-fasting state [55]. In addition, zinc concentrations are depressed by infection and inflammation [56]. Thus, plasma zinc concentrations will potentially overestimate the extent of zinc deficiency in the setting of infection or inflammation.

### Recommendations and Implications

1. Future studies into zinc deficiency following BS should examine zinc absorption and compare the degree of absorption in different types of BS.
2. More RCTs are needed to examine the effectiveness of zinc in taste change treatment following bariatric surgery to obtain more reliable results.
3. Studies to link taste change with zinc levels to weight loss or weight regain
4. More RCTs are required to examine the association between taste change and saliva flow rate, leading to more reliable results.
5. Time, dosage, and duration of supplementation need further investigation. RCTs into the effectiveness of zinc in taste change treatment used zinc supplementation of around 45–50 mg, 2 or 3 times per day, and found it effective in taste change recovery. However, in current clinical practice, patients still receive only between 8 and 15 mg of zinc supplementation.

## Conclusion

In conclusion, although causal link cannot be established, zinc deficiency appears to be linked with taste change following bariatric surgery. Supplementation with much higher doses of zinc—45–50 mg—has been effective in taste change treatment for a period of 3–6 months, for many cases of taste disorder. The results of this review are in agreement with the updated BOMSS guidelines that BS patients need high dose of zinc supplements and that zinc deficiency occurs most frequently after duodenal switch (DS). Yet routine multivitamin and mineral supplementation prescribed to patients following BS, in current clinical practice, are currently ineffective at avoiding zinc deficiency following BS. It is therefore also ineffective at avoiding taste change. Overall, taste change is more frequent after RYGB procedure comparing to other types of BS procedures. More research is crucial, to explore the association between zinc deficiency and taste change following BS—as well as the effect of BS in zinc absorption—and to ensure that BS patients are being given the level of zinc supplementation they require.

## Abbreviations

AGB: Adjustable gastric banding
BPD: Biliopancreatic diversion
BS: Bariatric surgery
DS: Duodenal switch
EWL%: Excessive weight loss percentage
GBP: Gastric bypass
LAGB: Laparoscopic adjustable gastric banding
LRYGB: Laparoscopic Roux-en-Y gastric bypass
LSG: Laparoscopic sleeve gastrectomy
NHS: National Health Service
RYGBP: Roux-en-Y gastric bypass
SG: Sleeve gastrectomy
BOMSS: British obesity and metabolic surgery society
